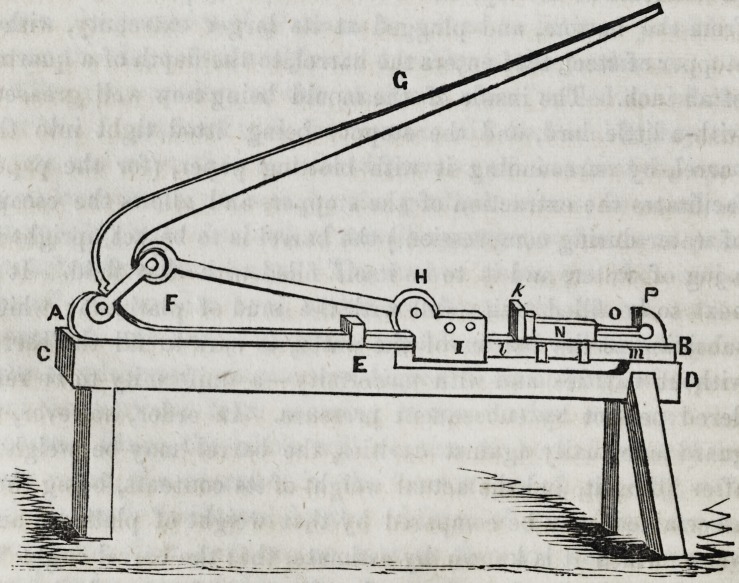# Chemistry of the Metals—Platinum

**Published:** 1854-01

**Authors:** Reginald N. Wright


AMERICAN JOURNAL

OF

### DENTAL SCIENCE.

Vol. IV. NEW SERIES—JANUARY, 1854. NO. 2.

### ARTICLE I.

*Chemistry of the Metals—Platinum.* By Professor REGINALD N. WRIGHT, A. M., M. D.

(Continued from page 14.)

THE discovery of this metal is of comparatively recent date, going back no farther than the year 1741; it was then first mentioned. It occurs only in the metallic state, and commonly in small masses or grains, which are sometimes flat, sometimes rounded, and always possessing a high metallic lustre. It was first discovered in the West Indies, and described by Dr. Wood; it was afterwards discovered in considerable quantities in the alluvial depositions of several of the South American states, and described by Boussingault and Humboldt; the latter philosopher, having been so fortunate as to discover a piece larger than a pigeon's egg, and weighing 1088.5 grains. Brazil, Colombia and Peru are the South American provinces yielding the largest amount of the metal. The metal from the province of Antioquia, and described by Boussingault, occurs in sienitic veins. At a period later than the date of its discovery in America, platinum has been found in considerable quantities in the Ural mountains. Its grains commonly contain *gold*, *irridium*, *palladium*, *rhodium* and *osmium;* occasionally some others.

Platinum is exceedingly refractory in the fire, being unaffected by the heat of strong wind furnaces, but fusing with scintillation in the flame of the oxyhydrogen blowpipe; it is malleable and ductile to an astonishing degree, and like iron, may be welded with a hammer. It resists the action of most acids and the air, and is quite indispensable in the operations of the laboratory.

With regard to the action of platinum on gasses, Turner has the following paragraph, (vol. i, page 603,) "The remarkable property, observed by Döbereiner, in *spongy platinum* of causing the union of oxygen and hydrogen gasses, was mentioned at page 251; a property, which Dubony and Thenard showed to be also possessed, though in a lower degree, by platinum in its compact form of wire or foil, and by several other metals. (Au. de Ch. et Ph. xxiii and xxiv.) Faraday (Phil. Trans. 1834, part i) has lately discussed, with his wonted ability and success, both the conditions required for the effective action of platinum, and the cause of the phenomenon. The sole conditions are, purity of the gasses, and perfect cleanliness of the platinum. By cleanliness is meant, perfect absence of foreign matter, pure water excepted; and this condition is easily secured, by *fusing pure potassa* on its surface, *washing off the alkali, by pure water*, then dipping the platinum in hot *oil of vitriol*, and again washing with water. In this state, platinum foil acts so rapidly at common temperatures on oxygen and hydrogen gasses mixed in the ratio of 1 to 2, that it often becomes red hot and kindles the mixture. Handling the platinum, wiping it with a towel, or exposing it to the atmosphere for a few days, suffices to soil the surface of the metal, and thereby diminish or prevent its action. These phenomena, are supposed to result from the concurring influence of two forces, the *self-repulsive* energy of similar gaseous particles, and the adhesive attraction exerted between them and the platinum. Each gas, repulsive to itself and not repelled by the platinum, comes into the most intimate contact with the metal, and both gasses are so condensed upon its surface, that they are brought within the sphere of their mutual attraction, and combine." He goes on to say, that "the action of the platinum is retarded or wholly destroyed by the presence of small quantities of certain *gasses*, such as *hydrosulphuric acid*, *carbonic oxyd*, and *olefiant gasses.* One would be tempted to suppose that these gasses act by soiling the metallic surface, though in some respects the explanation is not satisfactory."

We next proceed to lay before our readers, the method of purifying platinum, and inasmuch as the credit of the best process seems to be almost universally ascribed to Dr. Wollaston, we subjoin his plan as copied from the *Philosophical Transactions of* 1829.

"The usual method of giving chemical purity to this metal, by solution in *aqua regia*, and precipitation with *sal ammoniac*, are known to every chemist; but I doubt whether sufficient care is usually taken, to avoid dissolving the iridium, contained in the ore, by due dilution of the solvent. In an account which I gave in the *Philosophical Transactions of* 1804, of a new metal, *rhodium*, contained in crude platinum, I have mentioned this precaution, but omitted to state to what degree the acids should be diluted. I now, therefore, recommend, that to every measure of the strongest muriatic acid employed, there be added an equal measure of water; and, that the nitric acid used, be what is called '*single aquafortis ;*' as well for the sake of obtaining a purer result, as of economy in the purchase of *nitric acid.* With regard to the proportions in which the acids are to be used, I may say, in round numbers, that *muriatic acid*, equivalent to 150 marble, and *nitric acid*, equivalent to 40 marble, will take 100 of crude platinum; but in order to avoid waste, and render the solution purer, there should be in the menstruum, a redundance of 20 per cent. at least, of the ore. The acids should be allowed to digest three or four days, with a heat gradually raised. The solution, being then poured off, should stand until a quantity of fine pulverulent ore of *iridium*, suspended in the liquid, has subsided; and, should then be mixed with 41 parts of sal ammoniac, dissolved in about 5 times their weight in water. The first precipitate, which will thus be obtained, will weigh about 165 parts, and will yield about 66 parts of pure platinum. As the mother-liquor will still contain about 11 parts of platinum, these, with some of the other metals yet held in solution, are to be recovered, by precipitation, from the liquor with clean bars of iron, and the precipitate is to be redissolved in a proportionate quantity of *aqua regia*, similar in its composition to that above directed; but in this case, before adding *sal-ammoniac*, about 1 part by measure of strong muriatic acid should be mixed with 32 parts by measure, of the *nitro-muriatic* solution, to provent any precipitation of palladium, or lead, along with the ammonia-muriate of platinum. The yellow precipitate must be well washed, in order to free it from the various impurities, which are known to be contained in the complicated ore in question; and must ultimately be well pressed, in order to remove the last remnant of the washings. It is next to be heated with the utmost caution in a black-lead pot, with so low a heat as just to expel the whole of the sal ammoniac, and to occasion the particles of platinum to cohere as little as possible; for on this depends the ultimate ductility of the product.

"The gray product of the platinum, when turned out of the crucible, if prepared with due caution, will be found highly coherent, and must then be rubbed between the hands of the operator, in order to procure, by the gentlest means, as much as can possibly be so obtained, of metallic powder, so fine as to pass through a fine lawn sieve. The coarser parts, are then to be ground in a wooden bowl, with a wooden pestle, but, on no account with any harder material, capable of burnishing the particles of platinum; since every degree of burnishing, will prevent the particles from cohering in the further stages of the process. Since the whole will require to be well washed in clean water, the operator, in the later stages of grinding, will find his work facilitated by the addition of water, in order to remove the finer portions, as soon as they are sufficiently reduced, to be suspended in it.

"Those who would view this subject scientifically, should here consider, that as platinum cannot be fused by the utmost heat of our furnaces, and consequently cannot be freed, like other metals, from its impurities, during igneous fusion, by fluxes, nor be rendered homogenious by liquefaction, the mechanical division through water should here be made to answer, as far as may be, the purpose of melting; in allowing earthy matters to come to the surface by their superior lightness, and in making the solvent powers of water effect, as far as possible, the purifying powers of *borax* and *other fluxes*, in removing soluble oxyds.

"By repeated washing, shaking, and decanting, the finer parts of the gray powder of platinum may be obtained as pure, as other metals are rendered, by the various processes of ordinary metallurgy; and, if now poured over, and allowed to subside in a clean basin, a uniform mud or pulp will be obtained, ready for the further process of casting.

"The mould which I have used for casting, is a brass barrel $6\frac{3}{4}$ inches long, turned rather tapering within, with a view to facilitate the extraction of the ingot to be formed, being 1-12 inches in diameter at the top, and 1-23 inches, at a quarter of an inch from the bottom, and plugged at its larger extremity, with a stopper of steel, that enters the barrel, to the depth of a quarter of an inch. The inside of the mould being now well greased, with a little lard, and the stopper being fitted tight into the barrel, by surrounding it with blotting paper, (for the paper facilitates the extraction of the stopper, and allows the escape of water during compression,) the barrel is to be set upright in a jug of water, and is to be itself filled with that fluid. It is next to be filled quite full with the mud of platinum; which subsiding to the bottom of the water, is sure to fill the barrel without cavities and with uniformity—a uniformity to be rendered perfect by subsequent pressure. In order, however, to guard effectually against cavities, the barrel may be weighed after filling it, and the actual weight of its contents, being thus ascertained, may be compared by that weight of platinum and water, which it is known by estimate, that the barrel ought to contain. A circular piece of soft paper first, and then of woollen cloth, being laid upon the surface, allow the water to

16*

pass, during the partial compression by the force of the hand with a wooden plug. A circular plate of copper is then placed upon the top, and thus sufficient consistency is given to the contents to allow of the barrel being laid horizontally in a forcible press.

"The press which I have generally used for this purpose, consists of a flat iron bar A B, set edgeways, and screwed down by a hook E, near its middle, (where it would otherwise be liable to bend,) to a strong wooden bench C D. The bar is connected by a pivot at its extremity A, with the lever A, F, G. An iron rod F H, which turns at its two extremities upon the pivots, proceeds from the lever at F, and, as the lever descends, propels forward the carriage I, which slides along the bar. A stopper or block being placed in the vacant space I *k*, the carriage communicates motion to the cradle *k l m*, which is also made to slide along the bar, and carries the barrel N, which lies upon the cradle, straight against the piston O, which rests by its end against P, a projection in the further extremity of the bar.

"The weight, which in this machine, when the angle of the lever's elevation is small, will keep the power, applied vertically at the extremity of the lever, *in equilibrio* = that power $\times \frac{AG \times FH}{AF\,[AF+FH]} \times$ cotan. of the angle of the lever's elevation; which expression, in the case of the press actually used, becomes power $\times$ 5 cotan. of the angle of the lever's elevation. This expression, at an elevation of 5°, becomes nearly 60 $\times$ power, and at an elevation of 10°, becomes nearly 300 $\times$ power; and when the lever becomes horizontal, the multiplier of the power becomes quasi infinite. This explanation will be sufficient to show the mechanical advantage, with which, by means of this press, the weight of the operator, acting on the end of the lever, will be made to bear against the area of the section of the barrel, a circle little more than an inch in diameter. After compression, (which is to be carried to the utmost limit possible,) the stopper at the extremity being taken out, the cake of platinum will easily be removed, owing to the conical form of the barrel; and being now so hard and firm, that it may be handled without danger of breaking, it is to be placed upon a charcoal fire, and there heated to redness, in order to drive off moisture, burn out grease, and give to it a firmer degree of cohesion. It is next to be heated in a wind-furnace; and, for this purpose, is to be raised upon an earthen stand, about 2½ inches above the grate of the furnace, the stand being strewn over with a layer of clean quartz ore sand, on which the cake is to be placed, standing upright on one of its ends. It is then to be covered with an inverted cylindrical pot, of the most refractory crucible-ware, resting at its open end upon the layer of sand; and care is to be taken that the sides of the pot do not touch the cake. To prevent the blistering of the platinum by heat, (which is the usual defect of this metal in its manufactured state,) it is essential to expose the cake to the most intense heat that a wind-furnace can be made to receive, more intense than the platinum can well be required to bear under any subsequent treatment, so that all impurities may be totally driven off, which any lower temperature might otherwise render volatile. The furnace is to be fed with *coke*, and the action of the fire is to be continued for about twenty minutes from the time of lighting it, a breathing heat being maintained during the last four or five minutes. The cake is now to be removed from the furnace, and, being placed upright upon an anvil, is to be struck, while hot, on the top with a heavy hammer, so as, at one heating, effectually, to close the metal. If, in this process of forging, the cylinder should become bent, it should on no account be hammered on the side, by which treatment it would be cracked immediately; but must be straightened by blows upon the extremities, dexterously directed, so as to reduce to a straight line the parts which project.

"The work of the operator is now so far complete that the ingot of platinum may be reduced, by the process of heating and forging, like that of any other metal, to any form that may be required. After forging, the ingot is to be cleaned from the ferruginous scales, which its surface is apt to contract in the fire, by smearing over its surface with a moistened mixture of equal parts, by measure, of crystallized *borax* and *common salt of tartar*, (which, when in fusion, is a ready solvent of such impurities,) and then exposing it, upon a platina-tray, under an inverted pot, to the heat of a wind-furnace. The ingot, on being taken out of the furnace, is immediately to be plunged into dilute sulphuric acid, which, in the course of a few hours, will entirely dissolve the flux adhering to the surface. The ingot may then be flattened into leaf, drawn into wire, or submitted to any of the processes of which the most ductile metals are capable.

"The perfection of the methods above described, for giving to platinum complete malleability, will best be estimated by comparing the metal thus obtained, in respect of its specific gravity, with platinum which has undergone complete fusion; and by comparing it in respect of its tenacity, with other metals possessing that quality in the greatest perfection. The specific gravity of platinum, drawn into fine wire from a button which had been completely fused by the late Dr. E. D. Clarke, with an oxyhydrogen blow-pipe, I found to be 21·16. The aggregate specific gravity of the cake of metallic mud, when first introduced into the barrel, exclusively of moisture, is about 4·3; when taken from the press, it is about 10. That of the cake fully contracted, on being taken out of the wind-furnace before forging, is from 17 to 17·7. The mean specific gravity of the platinum, after forging, is about 21·25, although, that of some rods, after being drawn, is 21·4: but that of fine platinum-wire, determined by comparing the weight of a given length of it, with the weight of an equal length of gold wire, drawn through the same hole, I find to be 21·5, which is the maximum specific gravity that we can well expect to be given to platinum.

"The mean tenacity, determined by the weight required to break them, of two fine platinum-wires, the one of $\frac{1}{3000}$, the other of $\frac{1}{3850}$ of an inch in diameter, reduced to the standard of a $\frac{1}{10}$ of an inch in diameter, I found to be 409 pounds; and the mean tenacity of eleven wires, beginning with $\frac{1}{4500}$ and ending with $\frac{1}{25000}$ of an inch, reduced to the former standard, I found to be 589 pounds; the maximum of these eleven cases being 645 pounds, and the minimum 480 pounds. The coarsest and the finest wire which I tried, present exceptions, since a wire of $\frac{1}{150}$ of an inch gave 290 pounds, and a wire of $\frac{1}{30000}$ of an inch, 190 pounds. If we take 590 pounds, (as determined by the eleven consecutive trials,) to be the measure of the tenacity of the platinum prepared by the process above described, and consider that the tenacity of gold-wire, reduced to the same standard, is about 500, and that of iron-wire 600, we shall have full reason to be satisfied with the processes above detailed, by which platinum has been rendered malleable."

The account of the method of treating crude platinum we have given above, is (as we before remarked) that of Dr. Wollaston, as published in the Philos. Trans. of 1829, and we have taken the liberty of copying it verbatim, both because we believe it to be the best method extant, and because we could not describe it better, or more intelligibly, than he has done; the *careful observance* of the rules of practice therein laid down, will ensure a uniform and perfectly satisfactory result in every instance.

The following account of the alloys of platinum will be found in Brande, page 995:

"Alloys of Platinum.—With *potassium* and *sodium* it forms compounds which decompose water—(Davy.) Its alloy with *manganese* is unknown. *Iron* and *platinum* in equal parts, form a crystalline alloy which takes a fine polish. According to Dr. Lewis, the alloy of *cast iron* and *platinum* is hard, tough, and somewhat ductile, the density greatly exceeding the mean: it is brittle when hot. Stodart and Faraday found the toughness and smoothness of steel improved by one-hundredth of platinum—(Phil. Trans., 1822.) Wires of *steel* and *platinum*, when welded and polished, exhibit a curious and beautiful surface, especially when the steel parts are slightly acted on by dilute acid. This welding property of platinum may be usefully applied in the arts; wires may be joined so as to form rings and chains; and with a view to economy, platinum may be joined to iron or steel for many uses in the laboratory of the chemist. Platinum dissolves in fused zinc; the alloy is brittle, bluish-white, and hard: one-twentieth of platinum destroys the malleability of zinc, and one-fourth of zinc renders platinum brittle—(Lewis.) Tin and platinum combine in all proportions, forming alloys more or less brittle and fusible. When *tin-foil* and platinum are wrapped together, and heated by the blowpipe, they combine with incandescence—(Fox, Ann. Phil., xiii, 467.) The alloy of *cadmium* and platinum is white, granular, brittle and easily fusible: heated till the excess of cadmium is expelled, it contains 100 *platinum* + 117 *cadmium*—(Thomson.) The alloy of *cobalt* and *platinum* is comparatively fusible. With its weight of *nickel*, platinum forms a pale-yellow alloy, susceptible of a high polish, and obedient to the *magnet*. *Copper* and platinum form alloys, the ductility and color of which vary with the proportions. Platinum easily destroys the color of copper; this compound has been recommended for the mirrors of reflecting telescopes; an alloy of 7 platinum, 16 copper, 1 zinc, resembles gold in color—(Cooper, Quart. Jour., iii, 119.) *Lead* and *platinum* form brittle alloys, not entirely decomposed by cupellation—(Dumas.) *Antimony* forms a gray compound with *platinum*, partly decomposed by heat, and entirely by roasting; these metals enter into ignition when they combine, in the same manner as *tin* and *zinc*—(Fox.) *Bismuth* and *platinum* form brittle alloys, not entirely decomposed by cupellation—(Lewis.) *Arsenic* and *platinum* form a dark-gray, brittle alloy. When particles of arsenic are placed upon red-hot platinum-leaf, they immediately fuse a hole in it. When 2 parts of *platinum*, 2 of *arsenious acid*, and 1 of *potassa*, are fused together, a compound of 89 *platinum* and 10 of *arsenic* (1 atom of each,) is obtained: its density is 16.4; it is fusible at a red heat, but the whole of its arsenic cannot be expelled by heat. Equal parts of *molybdenum* and *platinum* melted into a hard, brittle mass: when the proportion of platinum was increased, the fusion was not complete—(Hielm.) *Mercury* amalgamates difficultly with *platinum;* a compound of 63 of *mercury* and 37 of *platinum* is a soft solid; *spongy platinum* forms the readiest combination; this amalgam adheres readily to the surface of glass. *Silver* and *platinum* form fusible and ductile alloys; when the silver predominates, they are soluble in nitric acid; by boiling sulphuric acid the silver only is dissolved. When the alloy is kept in fusion its components have a tendency to separate. *Gold* and *platinum* require a strong heat for combination, and the color of the gold is greatly deteriorated, even by one-twenty-second of platinum; an alloy of 4 of *platinum* and 1 of *gold*, nearly resembles *platinum* in color; the gold color does not predominate till it forms eight-ninths of the alloy—(Hatchet. Klaproth.)

[To be continued.]

### ARTICLE II.

*Mechanical Dentistry.* By JOHN LEWIS, D. D. S., Buffalo, N.Y.

## Figures and Tables

**Figure f1:**